# Long-Range Charge Transport Facilitated by Electron Delocalization in MoS_2_ and Carbon Nanotube Heterostructures

**DOI:** 10.1021/acsnano.4c12858

**Published:** 2025-01-15

**Authors:** Daria D. Blach, Dana B. Sulas-Kern, Bipeng Wang, Run Long, Qiushi Ma, Oleg V. Prezhdo, Jeffrey L. Blackburn, Libai Huang

**Affiliations:** † Department of Chemistry, Purdue University, West Lafayette, Indiana 47907, United States; ‡ Materials Science Center, National Renewable Energy Laboratory, Golden, Colorado 80401, United States; § Department of Chemical Engineering, University of Southern California, Los Angeles, California 90089, United States; ∥ College of Chemistry, Key Laboratory of Theoretical & Computational Photochemistry of Ministry of Education, Beijing Normal University, Beijing 100875, China; ⊥ Department of Chemistry, and Department of Physics and Astronomy, University of Southern California, Los Angeles, California 90089, United States

**Keywords:** two-dimensional semiconductors, carbon nanotubes, interfacial charge separation, mixed dimensional heterostructures, pump−probe microscopy

## Abstract

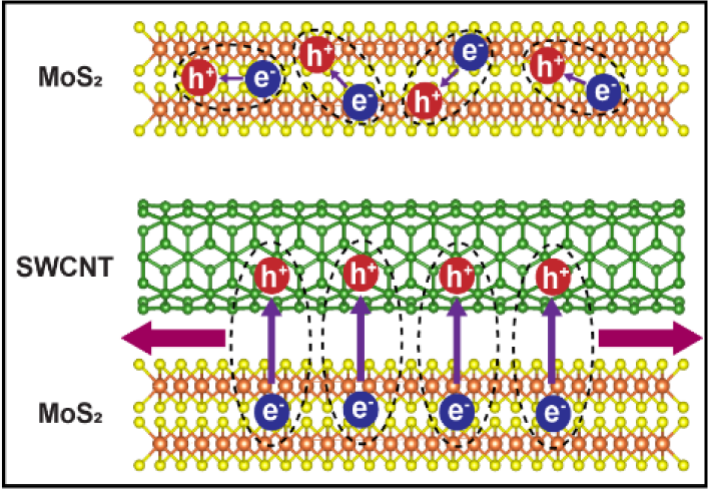

Controlling charge transport at the interfaces of nanostructures is crucial for their successful use in optoelectronic and solar energy applications. Mixed-dimensional heterostructures based on single-walled carbon nanotubes (SWCNTs) and transition metal dichalcogenides (TMDCs) have demonstrated exceptionally long-lived charge-separated states. However, the factors that control the charge transport at these interfaces remain unclear. In this study, we directly image charge transport at the interfaces of single- and multilayered MoS_2_ and (6,5) SWCNT heterostructures using transient absorption microscopy. We find that charge recombination becomes slower as the layer thickness of MoS_2_ increases. This behavior can be explained by electron delocalization in multilayers and reduced orbital overlap with the SWCNTs, as suggested by nonadiabatic (NA) molecular dynamics (MD) simulations. Dipolar repulsion of interfacial excitons results in rapid density-dependent transport within the first 100 ps. Stronger repulsion and longer-range charge transport are observed in heterostructures with thicker MoS_2_ layers, driven by electron delocalization and larger interfacial dipole moments. These findings are consistent with the results from NAMD simulations. Our results suggest that heterostructures with multilayer MoS_2_ can facilitate long-lived charge separation and transport, which is promising for applications in photovoltaics and photocatalysis.

Charge transport at heterointerfaces plays a key role in optoelectronic devices and solar energy harvesting applications. Transition metal dichalcogenides (TMDCs) are highly programmable two-dimensional (2D) materials that host strongly bound excitons with unique spin and valley properties.^1−5^ Heterostructures based on TMDCs show desirable properties associated with ultrafast charge transfer and the formation of interlayer excitons, which creates new opportunities for the development of optoelectronic and quantum information applications.^6−9^ The atomically thin and sharp interfaces of TMDCs allow subpicosecond rapid charge and energy transfer.^4,5,10−15^ More recently, hybrid-TMDC heterostructures combining other nanostructures have gained interest as a class of promising systems to achieve long-lived interfacial charge separation. In particular, semiconducting single-walled carbon nanotubes (SWCNTs) are versatile components to couple with TMDCs because of their high carrier mobilities, strong absorption at visible and near-infrared (NIR) wavelengths, high photochemical stability, and diameter-dependent tunability of their bandgaps.^16−19^

Recent studies of TMDC-SWCNT heterojunctions have reported microsecond-time scale charge separation following subpicosecond interfacial charge transfer,^20−22^ making these materials attractive for applications such as photovoltaics, photodetectors, and photocatalysis. For these applications, long-range transport of carriers at the interfaces is essential. However, despite the investigations of interfacial charge separation using ultrafast spectroscopy, a detailed understanding of how charge carriers migrate at the 2D-1D interfaces following charge transfer is still lacking. For instance, while charge transfer excitons are known to form at these interfaces,^23^ their migration mechanisms remain unclear. It has been hypothesized that the delocalization of charge transfer states plays a critical role in achieving effective charge separation.^24−26^ The atomically sharp interfaces and well-controlled layer thicknesses of TMDCs provide an opportunity to elucidate the role of charge delocalization. Direct imaging of charge transport at these interfaces with quantitative data and high spatial and temporal resolutions would be highly desirable.

In this work, we investigated charge separation and transport at the interfaces between a thin film of (6,5) SWCNTs and MoS_2_ layers by using transient absorption microscopy (TAM), which provides ∼200 fs temporal resolution and ∼50 nm spatial precision for imaging charge carriers and excitons. We demonstrate the ability to tune the time scale of charge separation and transport across the interfaces by varying the thickness of the MoS_2_ layer from a single layer to five layers. Our combined experimental and theoretical investigation reveals electron delocalization in multilayer MoS_2_ can facilitate long-lived charge separation at the interfaces. Additionally, charge separation induces an interfacial dipole moment that grows with layer thickness, leading to favorable long-range charge transport in heterostructures based on multilayer MoS_2_.

## Results and Discussion

### Charge Transfer and Recombination at the 1D–2D Interface

**Figure 1:**
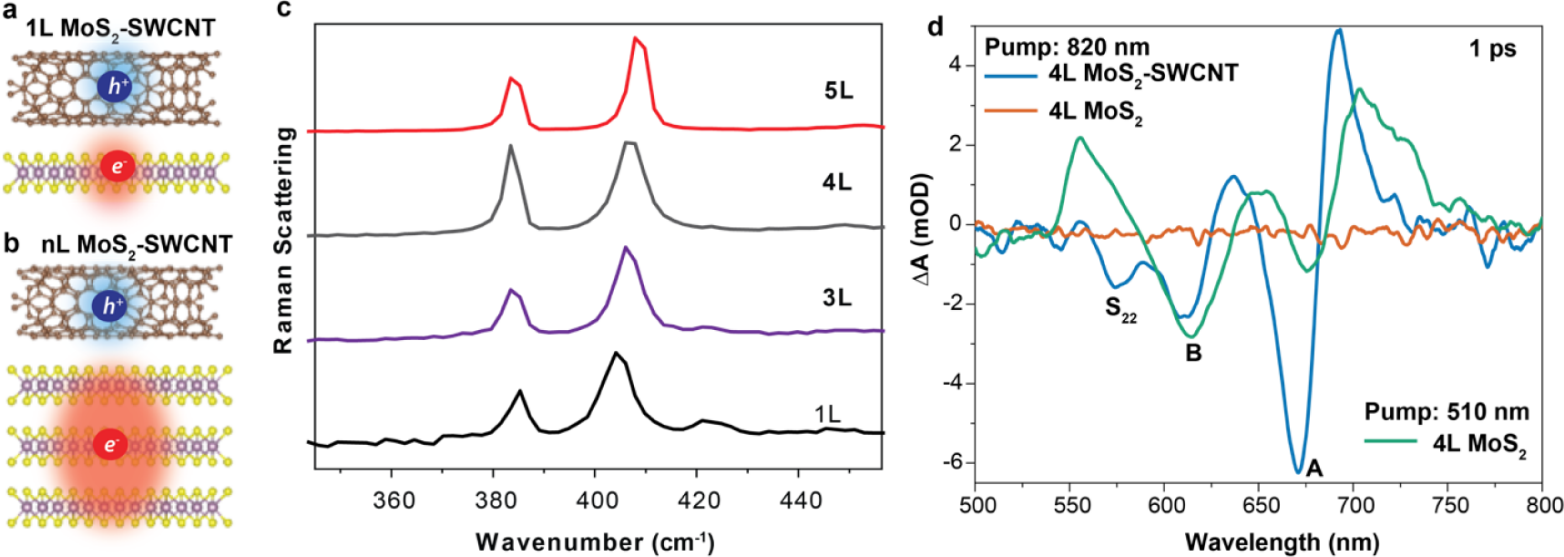
Ultrafast electron transfer in the MoS_2_–SWCNT heterostructures. (a) Schematic of the interfacial charge separation at the MoS_2_–SWCNT interfaces shown for 1L- and (b) multilayer (nL)-MoS_2_. (c) Raman spectra of 1L, 3L, 4L, and 5L MoS_2_. (d) Transient absorption spectra following 820 nm excitation of the 4L-MoS_2_–SWCNT heterostructure (blue) and neat 4L-MoS_2_ (orange), as well as transient absorption spectra following 510 nm excitation of neat 4L-MoS_2_ (green).

We prepared MoS_2_–SWCNT heterostructures with varying MoS_2_ layer thickness by spray coating a thin film (∼20 nm thick) of (6,5) SWCNT ink on glass substrates containing mechanically exfoliated MoS_2_ flakes (more details provided in the SI). Based on the conduction and valence band energies for single- (1L) and multilayered (nL) MoS_2_ as well as SWCNT,^27,28^ type-II heterostructures are expected, in which charge separation occurs at the interface with electrons and holes located in different layers, as illustrated in Figure 1a. The conduction band minima (CBM) of both single-layer and multilayer MoS_2_ lie lower than the CB of SWCNT, facilitating electron transfer from SWCNT to MoS_2_. The valence band maximum (VBM) of SWCNT is located higher than the VBs of MoS_2_ allowing for hole transfer from MoS_2_ to SWCNT. In heterostructures with nL-MoS_2_, the electrons become delocalized across the layers, potentially leading to a larger spatial separation between the electrons and holes. To investigate the impact of electron delocalization, we prepared MoS_2_–SWCNT heterostructures with 1, 3, 4, and 5 MoS_2_ layers for comparison. Using Raman spectroscopy, we identified the thickness of MoS_2_ layers based on the energy difference between in-plane () and out-of-plane (*A*_1*g*_) Raman peaks (Figure 1b).^29,30^ A typical absorption spectrum of MoS_2_–SWCNT heterostructures is shown in Figure S1.

We first employed broadband transient absorption (TA) spectroscopy to investigate charge transfer and recombination dynamics at the 1D–2D heterostructure interfaces as a function of the MoS_2_ layer thickness. First, we chose to selectively excite the SWCNTs in the heterostructure with an 820 nm pump pulse. This pump photon energy is not high enough to excite the MoS_2_ layer, as it is below the MoS_2_ bandgap energy. After photoexcitation, the electrons in the SWCNTs are expected to be transferred to the MoS_2_ layer across the interface. Figure 1c plots the TA signal intensity at 1 ps for a 4L-MoS_2_–SWNT heterostructure (additional time-dependent TA spectra in Figure S2). We observe bleaching at 575 nm, corresponding to the SWCNT S_22_ transition. In addition, there are bleach bands at 615 and 675 nm consistent with the MoS_2_ A and B excitons, respectively. The photoinduced bleach bands arise from electron transfer from SWCNT to the MoS_2_ layer, consistent with previous reports.^16,17,22^ In contrast, no clear spectrum was observed for the MoS_2_ only control sample without SWCNTs under the same experimental conditions exciting at 820 nm (orange line in Figure 1d). The prominent derivative line shape near the A exciton resonance can be attributed to the increased trion absorption after photon-induced electron transfer. For comparison, we measured a 4L-MoS_2_ only control sample with above-bandgap excitation at 510 nm that generates excitons. The bleach bands from the A and B excitons (shown as a green solid line in Figure 1d) were observed. These bands are broader and shifted compared to those with 820 nm excitation. The differences in spectral shape and peak position between the two pump wavelengths highlight how photoinjected electrons and bound excitons distinctly modify the absorption of MoS_2_. Specifically, electron transfer from the SWCNT in the heterostructure with 820 nm excitation causes a redshift in exciton transition via bandgap renormalization and potential trion absorption, resulting in a derivative-like line shape (blue solid line in Figure 1d). In contrast, exciton generation with 510 nm excitation in the control MoS_2_ sample primarily broadens the transition, producing photoinduced absorption features on both sides of the exciton bleach (green solid line in Figure 1d). Additionally, the presence of SWCNTs modifies the dielectric environment, which may also contribute to the observed peak shifts.

Figure S3 compares steady-state PL spectra of the 1L-MoS_2_–SWCNT heterostructure and the control 1L-MoS_2_ before the deposition of SWCNTs, with photoexcitation at 447 nm. The 1L-MoS_2_ shows an emission peak near 660 nm corresponding to the A exciton. However, under the same experimental conditions, the 1L-MoS_2_–SWCNT heterostructure shows no detectable PL signal in this spectral range. This quenching of the MoS_2_ A exciton emission in the heterostructure further confirms a type II band alignment at the interface.^20,21^ Such a quench in PL can be anticipated, resulting from the hole transfer from MoS_2_ to the SWCNT, coupled with a concurrent electron transfer in the opposite direction. As the layer thickness increases, multilayer MoS_2_ becomes an indirect bandgap semiconductor, and hence, PL was not observed.

**Figure 2:**
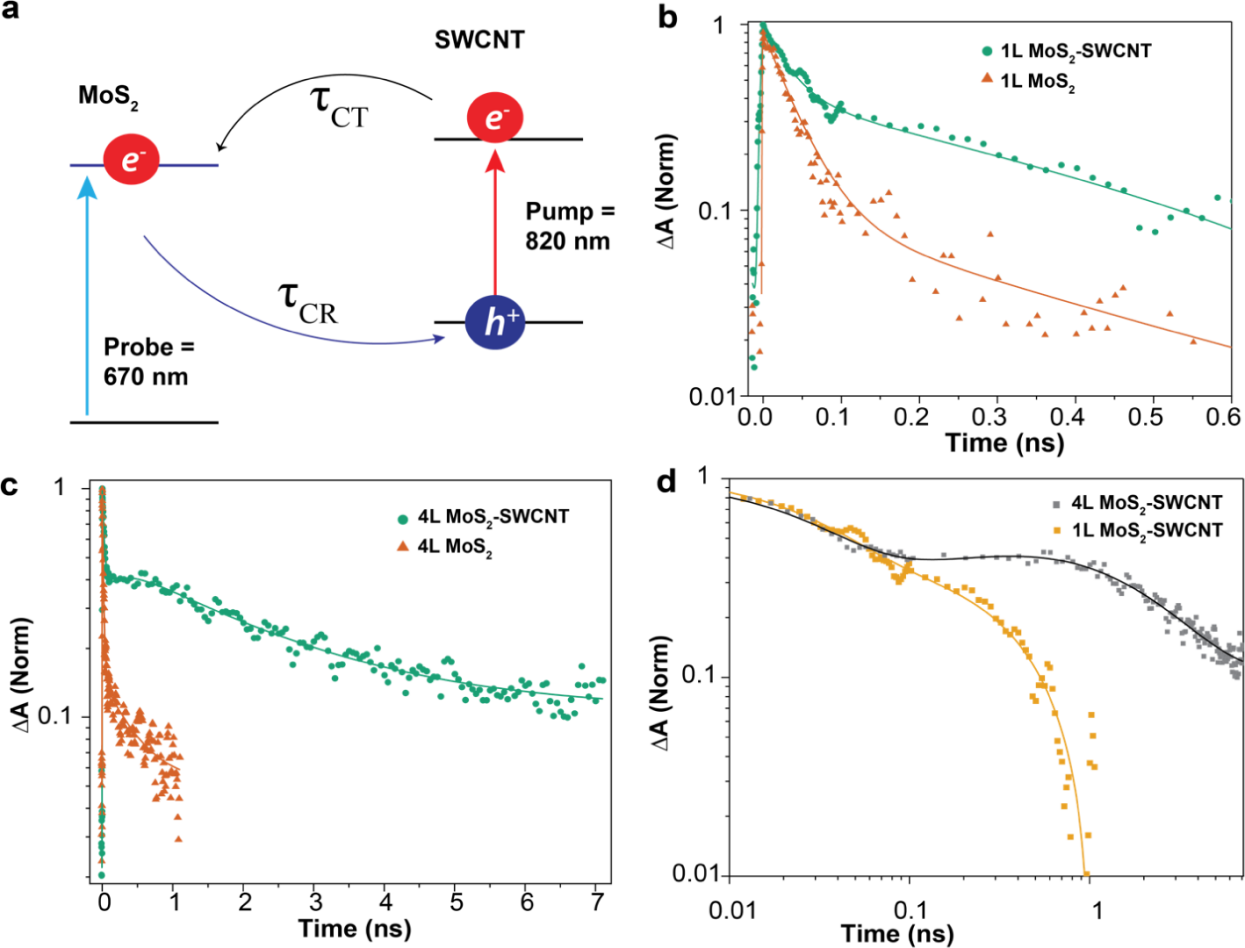
Charge separation and recombination dynamics in MoS_2_–SWCNT heterostructures. (a) Schematic illustration of charge transfer and recombination at the interface. *τ*_*CT*_: charge transfer time, *τ*_*CR*_: charge recombination time. (b) Carrier dynamics probed at 670 nm for the 1L-MoS_2_–SWCNT heterostructure (green) under 820 nm excitation and a controlled 1L-MoS_2_ (orange) under 630 nm excitation. (c) Carrier dynamics probed at 670 nm for the 4L-MoS_2_–SWCNT heterostructure (green) under 820 nm excitation and a controlled 4L-MoS_2_ (orange) under 630 nm excitation showing a long-lived charge separated state in the heterostructure. (d) Comparison of charge recombination in 1L-MoS_2_–SWCNT and 4L-MoS_2_–SWCNT heterostructures. The solid lines in panels (a)–(c) represent the results of a multiexponential decay fit, including contributions from charge separation and recombination. The details for the fitting can be found in Table S1.

Next, we examine charge transfer and recombination kinetics, as schematically illustrated in Figure 2a by probing the population of electrons in MoS_2_. To increase signal-to-noise, these measurements were carried out with a single-wavelength probe at the A exciton resonance at 670 nm. Charge transfer and recombination dynamics were found to depend strongly on the MoS_2_ thickness. The results demonstrate a long-lived charge-separated state in the heterostructures (exciting SWCNTs only at 820 nm) compared to A exciton recombination in 1L-MoS_2_ only (Figure 2b) and 4L-MoS_2_ only (Figure 2c) control samples. These charge-separated states are also much longer-lived than the exciton lifetimes of polymer-wrapped SWCNTs dispersed in solution (Figure S4). Additional results from 3L- and 5L-MoS_2_–SWCNT heterostructures are shown in Figure S5. The charge transfer time *τ*_*CT*_ was determined by the rise time of the MoS_2_ exciton bleach signal when SWCNTs were selectively excited at 820 nm. To extract *τ*_*CT*_ and *τ*_*CR*_, we fitted the bleach dynamics with a multiexponential function convoluted with a Gaussian response function (details provided in Table S1). In the 1L-MoS_2_–SWCNT heterostructure, the rise time was limited by instrumental resolution of approximately 200 fs. For nL-MoS_2_–SWCNT heterostructures, an additional slower rise time of around 200 ps was observed, as shown in Figures 2c,d and S6, attributed to a slower charge transfer at the interfaces. The slow decay is assigned to *τ*_*CR*_ in the heterostructures. In the nL-MoS_2_ heterostructures, we observed a fast initial decay of approximately 30 ps, which we attribute to ultrafast relaxation processes within the MoS_2_ layer itself. This behavior arises because nL-MoS_2_ layers undergo a transition to indirect semiconductors, leading to a small absorption at 820 nm.

The charge recombination *τ*_*CR*_ in the nL-MoS_2_–SWCNT heterostructures is much slower than in the 1L-MoS_2_–SWCNT. The *τ*_*CR*_ is measured to be 0.5 ± 0.2 ns for the 1L-MoS_2_–SWCNT. For 4L-MoS_2_–SWCNT, *τ*_*CR*_ is much longer with a short component of 2.0 ± 0.2 ns, while the long component is limited by the 7 ns time window of our experiments. Charge-separated states could have lifetimes as long as μs, as previously reported.^20^ This strong thickness dependence for charge transfer is consistent with observations in PbSe-quantum-dot- MoS_2_^31^ and MoS_2_–MoSe_2_ heterostructures.^32^ 3L-MoS_2_–SWCNT and 5L-MoS_2_–SWCNT have similar charge transfer and recombination dynamics as the 4L-MoS_2_–SWCNT.

### Ultrafast Imaging of Carrier Transport at the Interfaces

Next, we employed TAM to directly image carrier transport in MoS_2_–SWCNT heterostructures following electron transfer by fixing the pump beam and scanning the probe beam relative to the pump in both spatial and temporal domains.^33^ We excited the heterostructures with an 820 nm pump to selectively generate excitons in SWCNT and used a 670 nm probe to track how the electrons move in MoS_2_ following the interfacial charge transfer (more details in the SI). Although the charge transfer yield is approximately 34%,^21^ excitons in SWCNTs that do not undergo charge transfer do not contribute to the TAM signal. As discussed in detail below, due to reduced dielectric screening, electrons in MoS_2_ are likely bound to the holes left behind in the SWCNTs at early times after charge transfer, causing the electrons and holes in these charge transfer excitons to move together in the bound state.

At zero-time delay, the exciton population created by a Gaussian pump beam can be described by a spatial distribution of  with a variance . Carrier diffusion can lead to a broadened Gaussian distribution described by  with a variance of  at a later delay time *t*. For normal diffusion driven by population gradient, linear temporal dependence of  is expected with a time-independent diffusion constant *D* given by .^33^ Diffusive exciton transport was observed in the control MoS_2_ layers as shown in Figure S6 with diffusion constants ranging from 0.7 cm^2^ s^–1^ (1L) to 6.7 cm^2^ s^–1^ (5L). The increased diffusion constant for an increasing number of layers is consistent with observations in other TMDCs.^34^ While the exciton diffusion constant in individual SWCNTs can be as high as 10 cm^2^ s^–1^, charge and exciton diffusion in SWCNT thin films is typically much slower (<1 cm^2^ s^–1^), primarily due to slow hopping at the points of direct contact between the tubes.^35−39^

**Figure 3:**
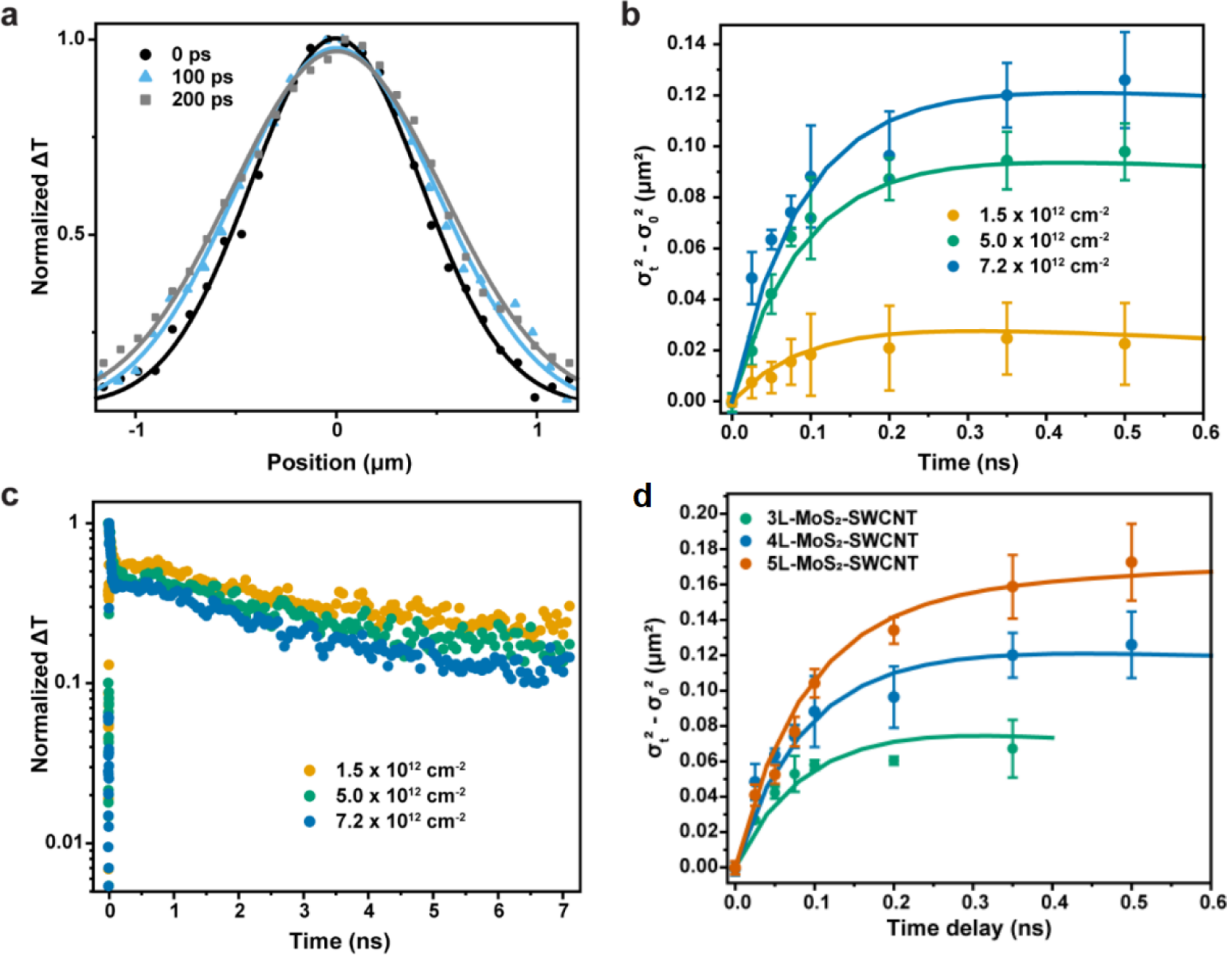
Charge transfer exciton interactions driving diffusion in MoS_2_–SWCNT heterostructures. (a) Carrier population profiles in the 4L-MoS_2_–SWCNT heterostructure fitted with Gaussian functions, with the maximum Δ*T* signal normalized, taken at 0, 100, and 200 ps time delay between the pump (820 nm) and probe (670 nm). The exciton density was fixed at 5.0 × 10^12^ cm^*–*2^. (b) Exciton density-dependent  as a function of delay time in the 4L-MoS_2_–SWCNT heterostructure. The solid lines are fits, using eq 2. (c) Exciton density-dependent carrier dynamics after 820 nm excitation of the 4L-MoS_2_–SWCNT heterostructure probed at 670 nm. (d) Thickness-dependent  as a function of delay time in the 3L-, 4L-, and 5L-MoS_2_–SWCNT heterostructures excited at 820 nm and probed at 670 nm with exciton density of 7.2 × 10^12^ cm^*–*2^. The solid lines are fits using eq 2.

Figure 3a shows the carrier population profiles at 0, 100, and 200 ps time delays for 4L-MoS_2_–SWCNT heterostructure (results for other heterostructures are shown in Figure S7). Interestingly, the transport in the heterostructures is strongly time-dependent, with a nonlinear increase in Gaussian variance in Figure 3b, which significantly deviates from the normal diffusion behavior seen in control MoS_2_ layers. Very rapid expansion was observed in the initial 200 ps, followed by much slower transport. In addition, carrier migration accelerates at higher densities. We examined the power-dependent carrier dynamics to ensure that the apparent fast transport did not result from higher-order processes such as Auger recombination or exciton–exciton annihilation.^40^ As shown in Figures 3c and S8, no significant change in the carrier recombination dynamics was observed as a function of exciton density after we subtracted the initial fast decay.

The density-dependent transport in MoS_2_–SWCNT heterostructures is indicative of drift-diffusion transport due to dipolar repulsion between interfacial charge-transfer excitons, similar to what was observed in WS_2_/WSe_2_ heterostructures in our recent report.^41^ Following photoexcitation, bound interfacial CT excitons are likely formed at the MoS_2_–SWCNT interfaces as a result of the reduced dielectric screening at the interfaces of TMDCs and SWNTs. Indeed, interlayer excitons have been reported in WSe_2_–SWCNT heterostructures^18^ as well as 1D van der Waals heterostructures of carbon nanotubes/BN/MoS_2_.^42^ The dipolar interaction between the interfacial CT excitons and their aligned dipole moments is net repulsive.^41^ As density increases, the repulsive interactions enhance carrier transport with an additional drift motion. To quantify the extent of motion of the carriers in the heterostructures, we employ a drift-diffusion model that has been previously used to describe similar anomalous diffusion in TMDC heterostructures.The rate equation that describes the spatial and temporal distribution of carriers is given by
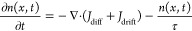
2where τ is the carrier lifetime. *J*_diff_ describes the normal diffusion resulting from the population gradient given by *J*_diff_ = −*D*∇*n*(*x,t*). *J*_drift_ denotes the drift flux due to the repulsive interactions between charges and can be described by *J*_drift_ = −*n*(*x,t*)*μu*_0_∇*n*(*x,t*), where μ is the exciton mobility and *u*_0_ is the interaction energy per unit density.^41,43^ The exciton interaction energy is the sum of the direct interaction energy (dipole–dipole) and the exchange interaction energy. The model effectively represents density- and time-dependent carrier transport in the 4L-MoS_2_–SWCNT heterostructure. For the 4L-MoS_2_–SWCNT heterostructure, we estimate the interaction energy to be approximately 1.15 × 10^–14^ eV/cm^2^. Recent studies on WSe_2_/WS_2_ heterostructures have reported a comparable interaction energy of about 1.6 × 10^–14^ eV/cm^2^.^32^ Since the exchange interaction scales inversely with distance,^44^ the differences in the interaction energies between the SWCNT-MoS_2_ system and WSe_2_/WS_2_ heterostructures could be caused by the latter having a stronger exchange interaction due to the small interlayer separation arising from two atomically thin layers of WSe_2_ and WS_2_.

Remarkably, at exciton densities of 5 × 10^12^ cm^*–*2^ and 7.2 × 10^12^ cm^*–*2^, transport in the 4L-MoS_2_–SWCNT heterostructure is enhanced over exciton transport in the 4L-MoS_2_ due to the strong repulsive interaction of the interfacial CT exciton (Figure S9) At a lower exciton density of 1.5 × 10^12^ cm^*–*2^, the transport of CT excitons in the 4L-MoS_2_–SWCNT appears to be lower, compared to the excitons in 4L-MoS_2_. One reason for this lower diffusion in heterostructures could be the presence of interfacial energy fluctuations, causing barriers in charge transport. The repulsive interaction at high density provides the energy driving force to overcome the energetic fluctuations at higher densities^41^

In addition to 4L-MoS_2_–SWCNT heterostructure, we also examined carrier diffusion behavior in 3L- and 5L-MoS_2_–SWCNT heterostructures, with data shown in Figure 3d and Figure S10. Both heterostructures exhibit similar nonlinear diffusion with a strong dependence on pump fluence in the sub-nanosecond time scale. Notably, charge transport at a given exciton density is enhanced as the layer thickness increases. The enhanced transport can be understood by that the thicker MoS_2_ layers should have larger interfacial dipole moments that lead to stronger repulsive interactions at interfaces. We also used eq 2 to model the transport and estimate the interaction energy, which was 0.73 × 10^–14^ eV/cm^2^ for the 3L-MoS_2_–SWCNT heterostructure and 1.25 × 10^–14^ eV/cm^2^ for the 5L-MoS_2_–SWCNT heterostructure. In the time scale beyond 300 ps, the transport is independent of exciton density, likely due to the diffusion of free charge carriers after the dissociation of CT excitons.

### Quantum Dynamics Simulations of Interfacial Charge Transfer

**Figure 4:**
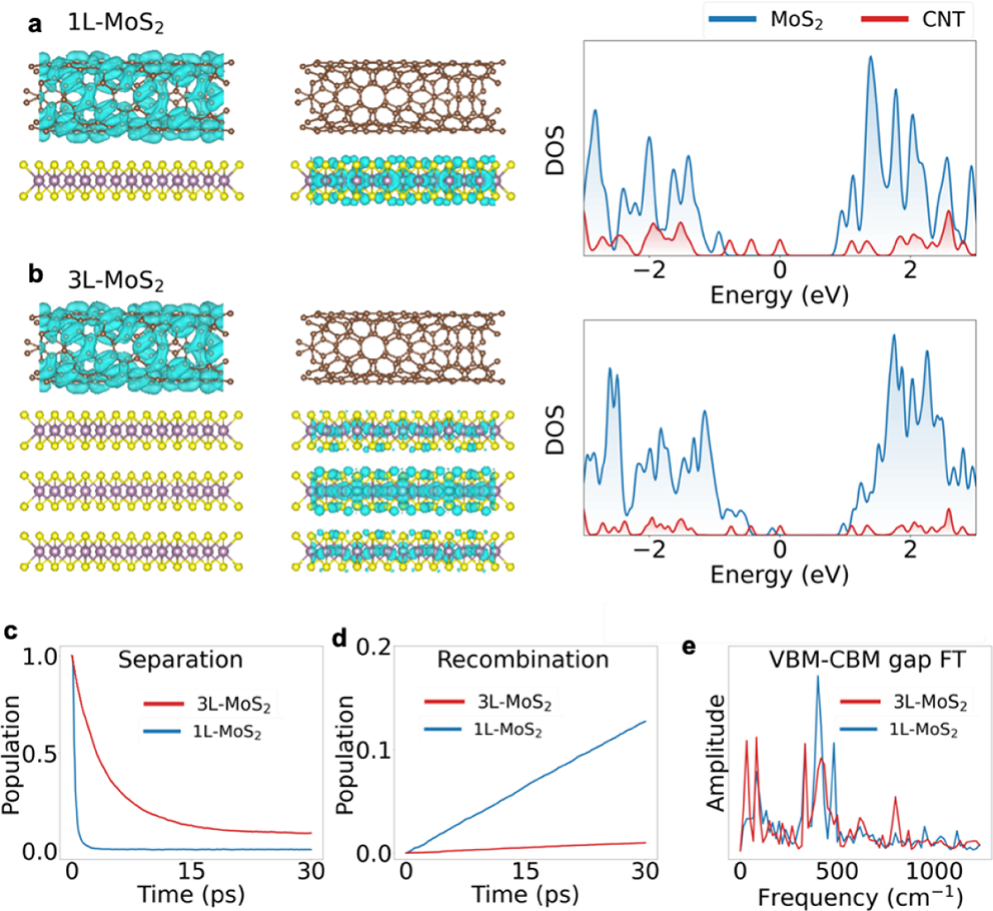
VBM (left) and CBM (right) charge densities and DOS of the SWCNT interfaced with (a) 1L-MoS_2_ and (b) 3L-MoS_2_. In both systems, the positive charge (VBM) is localized in the SWCNT, while the negative charge (CBM) is in MoS_2_. 3L-MoS_2_ shows proportionally higher peaks in DOS than 1L-MoS_2_, relative to the SWCNT contribution. The CBM is delocalized over all three layers of 3L-MoS_2_, with the most charge seen in the middle layer. The delocalization of the CBM leads to a large excited-state dipole moment, as shown in Table S1, in agreement with experiment. (c–d) Results of NAMD simulations for charge (c) separation and (d) recombination, corresponding to the CT and CR processes in Figure 2a. The time scales are reported in Table S1. More detailed data are listed in Figure S10. (e) Fourier transform (FT) of phonon-induced fluctuation of the VBM-CBM energy gaps (VBM-CBM gap FT) in the two systems. The 3L system exhibits more signals in the low frequency range than the 1L system.

To provide atomistic insights into the charge separation, transport, and recombination dynamics in the heterostructures involving SWCNTs interfaced with single and multilayer MoS_2_, we performed nonadiabatic (NA) molecular dynamics (MD) simulations on heterostructures with 1L- and 3L-MoS_2_. The simulation details are provided in the SI. The structures, charge densities of the VBM and CBM, and the electronic densities of states (DOS) are shown in Figures 4 and S11. The calculations verified that the MoS_2_–SWCNT interfaces form type II heterostructures, with the hole (VBM) localized in the SWCNT and the electron (CBM) residing in the MoS_2_.

The photoexcitation was modeled by promoting an electron from the SWCNT VBM to the SWCNT CBM, as shown schematically in Figure 2a. The calculations show that MoS_2_ has a significantly higher DOS than the SWCNT (Figure 4a,b), favoring the rapid transfer of photoexcited electrons from the SWCNTs into MoS_2_. In the 3L-MoS_2_ heterostructure, electrons spread over all layers, but the highest charge density is in the middle layer, with the layer next to the SWCNT having a lower density. Note the absence of charge density on sulfur atoms of MoS_2_ next to the SWCNT in the middle panel of Figure 4b. This contrasts with the corresponding charge density in 1L-MoS_2_, which shows significant localization on MoS_2_ sulfur atoms next to the SWCNT, in the middle panel of Figure 4a. The decreased overlap of the SWCNT and MoS_2_ orbitals in the 3L system compared to 1L-MoS_2_ suggests slower charge transfer and longer-lived charge separation with an increasing number of MoS_2_ layers.

Indeed, the NAMD simulations demonstrate that both photoinduced charge transfer and recombination are slower in thicker MoS_2_ layers, in qualitative agreement with experimental observations (Figures 4c,d, S12 and Table S2). We note, however, that NAMD simulations are computationally demanding, making it infeasible to match the exact time scale of the experimental measurements. The NA coupling responsible for charge recombination is several times smaller in the 3L system than in the 1L system (0.0763 vs 0.22 meV), due to the reduced overlap of donor and acceptor orbitals. The predicted charge separation and recombination times are in qualitative agreement with the experiments. The simulations also show that both charge separation and recombination are accompanied by the transfer of electronic energy to vibrations. Figures 4e and S13 report electron-vibrational influence spectra, computed as Fourier transforms of the phonon-induced fluctuations of the electronic energy levels. The influence spectra demonstrate that vibrations around 400 cm^–1^ are primarily responsible for the electron-vibrational energy exchange, and that the multilayer system exhibits stronger signals in the low-frequency range, 100 cm^–1^ and below.

The NAMD simulations also provided an understanding of the electron–hole distance and the dipole moment of the interfacial CT excitons. The charge-separated state generated by the photoexcitation greatly increases the dipole moment of the interface compared to the excitons in the MoS_2_ layers only. As the thickness of the MoS_2_ layer increases, the electron–hole distance increases, resulting in more delocalized charge transfer excitons. The dipole moment of the charge transfer state is 2.75 times larger in the 3L than in the 1L heterostructures (Table S2). In the presence of multiple excitations, the dipole moments are aligned and repel each other, favoring enhanced charge transport in the heterostructures with multilayer MoS_2_, as shown in Figure 3d. The large electron–hole distance also decreases the binding energy of the CT excitons, thereby facilitating their dissociation into free charge carriers.

## Conclusions

Interfacial charge transport is crucial for solar energy harvesting in excitonic nanostructures. In this study, we investigate charge transport in single-layer and multilayer MoS_2_-SWCNT heterostructures using a combination of ultrafast microscopy and first-principles theoretical calculations. By utilizing MoS_2_ with varying layer thicknesses, we elucidate the role of electron delocalization in interfacial charge transport. Electron delocalization is known to play a critical role in charge separation and generation in organic solar cells. For instance, with fullerene acceptors, increased electron–hole distance due to electron delocalization can lead to favorable charge dissociation by reducing orbital overlap.^24,25^ Consistent with these findings, we observe that the more delocalized CT states in multilayer MoS_2_ heterostructures result in longer-lived charge-separated states.

Our results also show that within the first 200 ps, the increased permanent dipole moment of the CT exciton induces dipolar repulsion, further enhancing interfacial charge transport. This nonlinear diffusion is strongly dependent on the MoS_2_ layer thickness. Specifically, as the interfacial CT excitons become more delocalized, the repulsive interaction between them increases, facilitating long-range diffusion. Thus, we suggest that heterostructures with multilayer TMDCs may be particularly advantageous for solar energy applications, offering both long-lived charge separation and long-range charge transport due to electron delocalization.

## Methods/Experiments

### Material Preparation

#### (**6**,**5**) SWCNT/PFO-BPy Ink

SWCNTs were prepared following a previously reported method.^20,21^ SWCNTs (CoMoCAT SG65i, CHASM) were added in a concentration of 0.5 mg/mL to a solution of 2 mg/mL poly-[(9,9-dioctylfluorenyl-2,7-diyl)-*alt*-co(6,6’-[2,2’-bipyridine])] (PFO-BPy, American Dye Source) in toluene. The SWCNTs were dispersed using a Cole-Parmer CPX 750 tip sonicator with a 0.5 in. diameter at 40% intensity for 15 min in a bath of flowing water. The dispersion was centrifuged for 5 min at 13,200 rpm and 20 °C in a Beckman Coulter L-100 XP ultracentrifuge with an SW 32 Ti rotor to generate a compacted pellet and supernatant rich in (6,5) SWCNTs. The pellet was discarded, and the supernatant was centrifuged for an additional 16 h at 24,100 rpm and 20 °C to generate a compacted pellet of (6,5) SWCNTs and supernatant containing excess PFO-BPy polymer. The supernatant was removed, and the (6,5) SWCNT pellet was gently rinsed with toluene before being redispersed in toluene by using a heated ultrasonic bath sonicator. The polymer removal centrifugation and subsequent redispersion were repeated twice.

#### Exfoliation of MoS_2_ Layers

Monolayer and few-layer MoS_2_ samples were mechanically exfoliated from bulk crystals (purchased from 2D Semiconductors) onto glass substrates. The number of MoS_2_ layers was determined by using Raman spectroscopy.

#### MoS_2_/SWCNT Heterostructures

MoS_2_/SWCNT heterostructures were prepared by spray coating the SWCNT ink onto MoS_2_ substrates, and neat films were prepared by spray coating onto glass substrates that were cleaned by sequential sonication in acetone and isopropyl alcohol.^20,21^ The substrates were placed onto a motorized metal stage that was heated to 130 °C to evaporate the solvent during the deposition. Prior to spray coating, the (6,5) SWCNT/PFO-BPy ink was diluted in toluene to reach an optical density of approximately 2.0 at the 1000 nm SWCNT S_11_ absorption peak. The ink was loaded into a syringe and injected at a rate of 0.3 mL/min through a SonoTek ultrasonic spray tip operated at 0.8 W. The ink mist was directed toward the substrates by N_2_ at a flow rate of 7.0 standard liters per minute, and the substrates were rastered beneath the mist to achieve 20–30 coats. The resulting films were soaked in toluene at 78 °C for 10 min to remove excess polymer.

### Optical Characterization

#### PL Spectroscopy

Steady-state optical measurements and time-resolved PL measurements were performed with a home-built confocal micro-PL setup.^9^ Samples were excited using a 447 nm picosecond-pulsed diode laser (LDH-P-C-450B, PicoQuant, fwhm of 50 ps, repetition rate 40 MHz). A 40× objective with a numerical aperture (NA) of 0.6 was used to both excite the sample and collect the sample emission. The PL emission was dispersed with a monochromator (Andor Technology) and detected by a thermoelectrically cooled charge-coupled device (CCD, Andor Technology).

#### Raman Spectroscopy

Measurements were conducted using an inVia Renishaw confocal Raman microscope equipped with a 532 nm laser. The excitation beam was focused through a 50× objective lens (NA = 0.75), and the Raman scattered light was collected using the same lens. The beam size during Raman measurements was approximately 1 μm, which is significantly smaller than the sample size of around 5 μm.

#### TA Spectroscopy

TA spectra were measured by a femtosecond pump–probe system with a home-built transient absorption spectrometer, as described in a previous publication.^45^ The output of a high repetition rate amplifier (PHAROS Light Conversion Ltd., 200 fs full width half-maximum, 750 kHz repetition rate, pulse energy of 20 μJ, 1030 nm fundamental) was split into two parts. One part was fed into an optical parametric amplifier (OPA, TOPAS-Twins, Light Conversion Ltd.) to generate 400 and 820 nm pump beams. The white light continuum (450–850 nm) was obtained by focusing the remaining part onto a Yttrium Aluminum Garnet (YAG) crystal. An optical chopper (MC200B, Thorlabs) was used to modulate the pump beam with a frequency of 195 Hz. Both pump and probe beams were focused on the sample and spatially overlapped. The probe light was collected by a lens and focused onto an array detector (Exemplar LS, B&W Tek). For transient dynamics scans, the probe beam was delayed relative to the pump beam by a linear stepper motor stage (ILS100PP, Newport).

#### TAM

Carrier transport was imaged by a home-built TAM setup.^40^ The output of a high-repetition-rate amplifier (Pharos Light Conversion, 750 kHz, 1030 nm) was pumped into two independent OPAs, one providing the pump and the other supplying the probe. Both the pump and probe beams were spatially filtered. An acousto-optic modulator (R23080-1, Gooch and Housego) was used to modulate the pump beam at 100 kHz. A mechanical translational stage (DDS600-E, Thorlabs) was used to delay the probe, with respect to the pump beam. Both the pump and probe beams were focused onto the sample by an objective (60×, NA 0.95). The probe beam was collected by another objective (Nikon, S Plan Fluor ELWD 20×, NA 0.45) and was detected by an avalanche photodiode (C5331-04, Hamamatsu). The change in the probe transmission induced by the pump was detected by a lock-in amplifier. A pair of galvanometer mirrors (GVS012, Thorlabs) was used to scan the beams to obtain carrier propagation images.

### Quantum Molecular Dynamics Simulations

#### Simulation Details

The ab initio DFT calculations were performed with the Vienna Ab initio Simulation Package (VASP).^46,47^ The Perdew–Burke–Ernzerhof (PBE) density functional^48^ and projected augmented pseudopotentials^49^ were used. The van der Waals interactions were treated by the DFT-D3 method with the Becke-Johnson damping function.^50^ The dipole correction was implemented to accurately calculate the dipole moments of the periodic systems. The cutoff energy of the plane wave basis was set to 400 eV. The convergence for the energy and the forces was 10^–4^ eV and 10^–3^ eV/Å, respectively.

Large simulation cells consisting of 260 and 476 atoms were employed, respectively, to model one-layer (1L) and three-layer (3L) MoS_2_ interfaces with the unit cell of the (6,4) SWCNT. The calculations employ the (6,4) SWCNT rather than the (6,5) SWCNT as in the experiments due to computational limitations. In particular, the unit cell of the (6,5) SWCNT contains 364 atoms, in comparison with 152 atoms of the (6,4) SWCNT unit cell. Both (6,4) SWCNT and MoS_2_ are periodic, and their lattices are not fully commensurate. There exists a 3% mismatch in their periodicities along the SWCNT direction, creating a 1.5% strain within each subsystem in this simulation. The structures were visualized with the VESTA software package, Figure S5. Starting with the experimental crystal structures, the system geometries were optimized, and then the systems were heated to 300 K by repeated velocity rescaling with a 1 fs atomic time step. Subsequently, 2 ps molecular dynamics (MD) trajectories were generated in the microcanonical ensemble.

The nonadiabatic (NA) MD simulations were performed using the Pyxaid software.^51,52^ The NA coupling matrix elements were computed numerically through the evaluation of wave function overlap at successive MD timesteps, adapted for the PAW method.^53,54^ The NAMD simulations were performed using the decoherence-induced surface hopping (DISH) approach,^55^ as implemented in the Pyxaid software.^52^ The pure-dephasing times were estimated via the second-order cumulant approximation of the optical response theory.^56^ The photoexcitation was modeled by promoting an electron from the SWCNT VBM to the SWCNT CBM, as shown schematically in Figure 2a. A total of 100 initial geometries were sampled for each system, and 1000 realizations of the stochastic DISH process were generated for each initial geometry.

## Supporting Information Available

Time-dependent TA spectra of ML-MoS_2_ samples; optical characterization of SWCNT excited state with TA; thickness-dependent electron transfer across heterostructures; additional diffusion data on 3L- and 5L- heterostructures (PDF)
