# Supplementary material for: Long-Range Charge Transport Facilitated by Electron Delocalization in MoS2 and Carbon Nanotube Heterostructures
Source: ACS Nano. 2025 Jan 15;19(3):3439–47. doi: 10.1021/acsnano.4c12858 (PMC11781022; doi:10.1021/acsnano.4c12858)
Supplement: Supplementary file 1 — nn4c12858_si_001.pdf [file nn4c12858_si_001.pdf]

Supporting Information for

Long-Range Charge Transport Facilitated by Electron Delocalization in Heterostructures of MoS_2 and Carbon Nanotubes

Daria D. Blach,[1] Dana B. Sulas-Kern,[2] Bipeng Wang,[3] Run Long,[4] Qiushi Ma[1],
Oleg V. Prezhdo,[5] Jeffrey L. Blackburn,[2] and Libai Huang[1]*

[1] Department of Chemistry, Purdue University, West Lafayette, Indiana, United States

[2] Materials Science Center, National Renewable Energy Laboratory, Golden, Colorado, United States

[3] Department of Chemical Engineering, University of Southern California, Los Angeles, CA 90089, USA

[4] College of Chemistry, Key Laboratory of Theoretical & Computational Photochemistry of Ministry of Education, Beijing Normal University, Beijing, 100875, People's Republic of China

[5] Department of Chemistry, and Department of Physics and Astronomy, University of Southern California, Los Angeles, CA 90089, USA

*Corresponding author: libai-huang@purdue.edu

Supplementary Figures

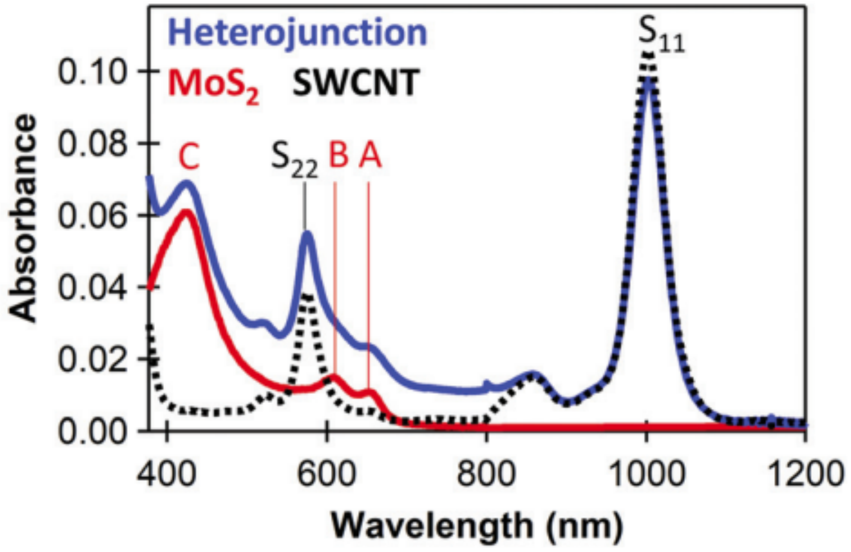

Fig. S1. A typical absorption spectrum of MoS_2-SWCNT heterostructures. Reproduced from Ref. 20, Sulas-Kern, D. B.; Zhang, H.; Li, Z.; Blackburn, J. L. Microsecond Charge Separation at Heterojunctions between Transition Metal Dichalcogenide Monolayers and Single-Walled Carbon Nanotubes. *Mater. Horiz.* 2019, *6*, 2103-211, with permission from the Royal Society of Chemistry. Copyright 2019.

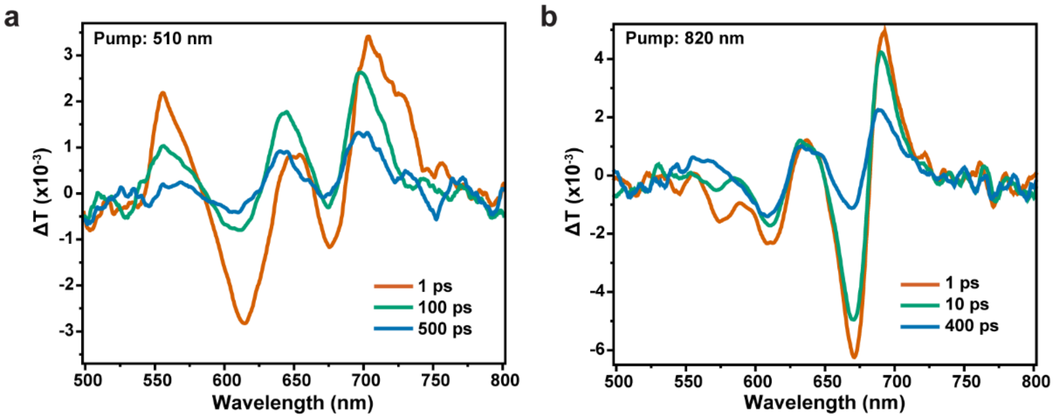

Fig. S2.
Time-dependent TA spectra of ML-MoS_2 samples. (a) Neat 4L-MoS_2 excited at 510 nm. (b) 4L-MoS_2-SWCNT excited at 820 nm.

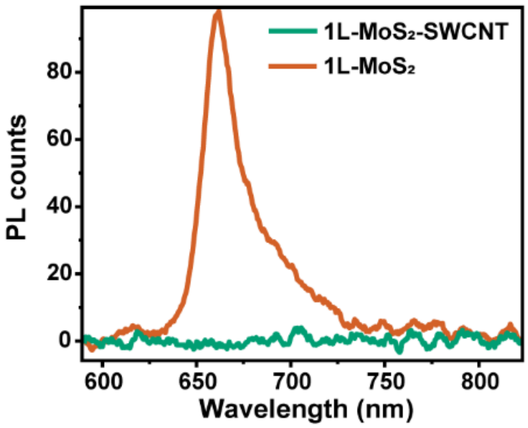

Fig. S3.
PL spectra for 1L-MoS_2 (orange) and the 1L-MoS_2-SWCNT heterostructure (green).

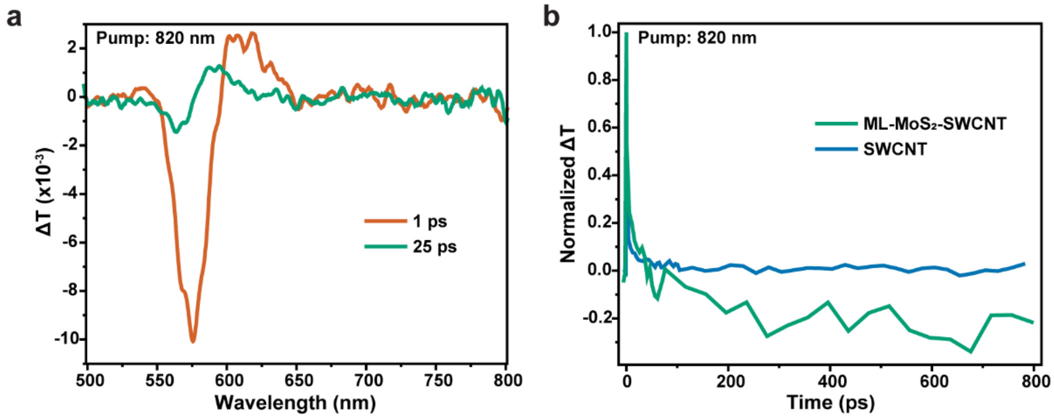

Fig. S4.
Optical characterization of SWCNT with TA. (a) TA spectra of polymer-wrapped SWCNT excited with 820 nm collected at 1 ps (orange) and 25 ps (green). (b) Transient dynamics of SWCNT S_{22} transition at 575 nm in the heterostructure (green) and neat sample (blue).

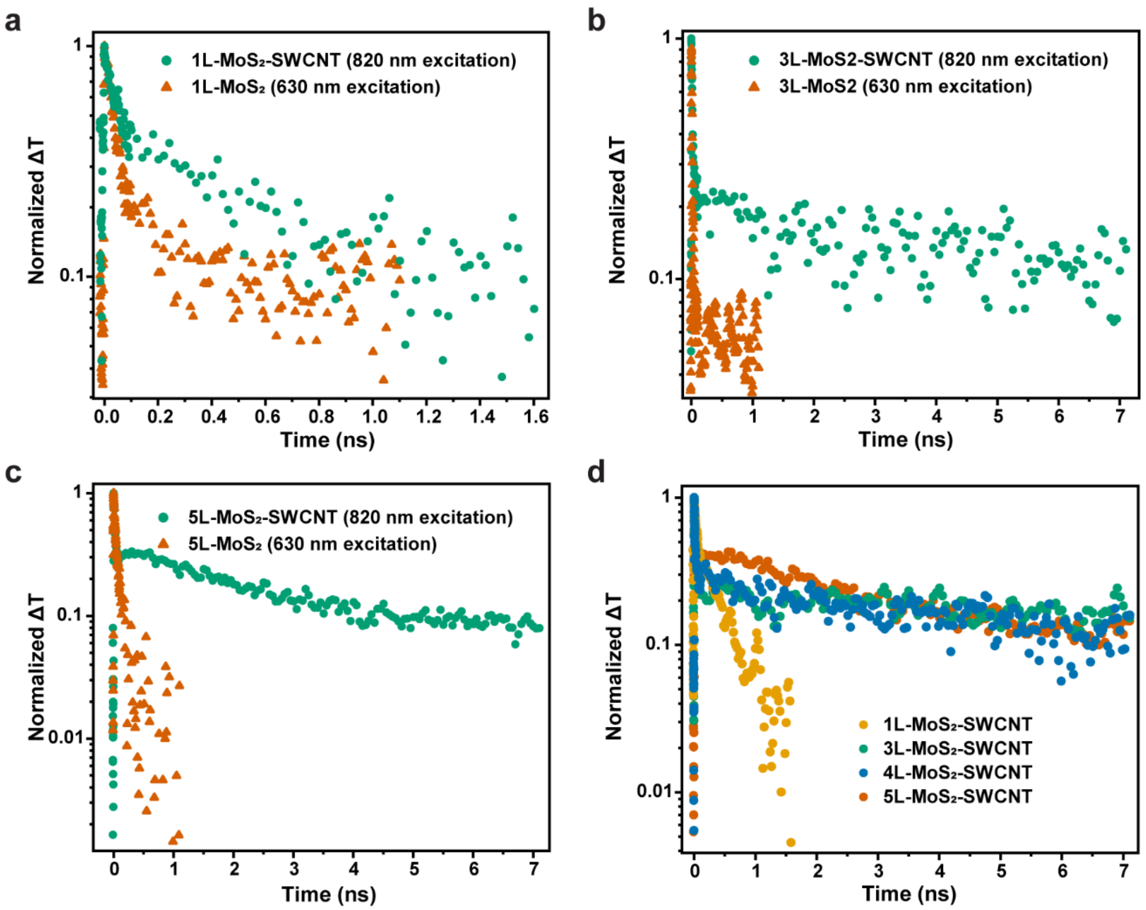

Fig. S5.

Thickness-dependent electron transfer across MoS_2-SWCNT heterostructures. TA dynamics probed at the A exciton bleach of the neat MoS_2 and MoS_2 in a heterostructure for (a) 1L-, (b) 3L-, and (c) 5L- MoS_2. Green dots represent the dynamics for heterostructures when excited at 820 nm and orange dots represent dynamics for neat MoS_2 samples excited at 630 nm. (d) Comparison of dynamics for 1L-, 3L-, 4L-, and 5L-MoS_2-SWCNT heterostructure.

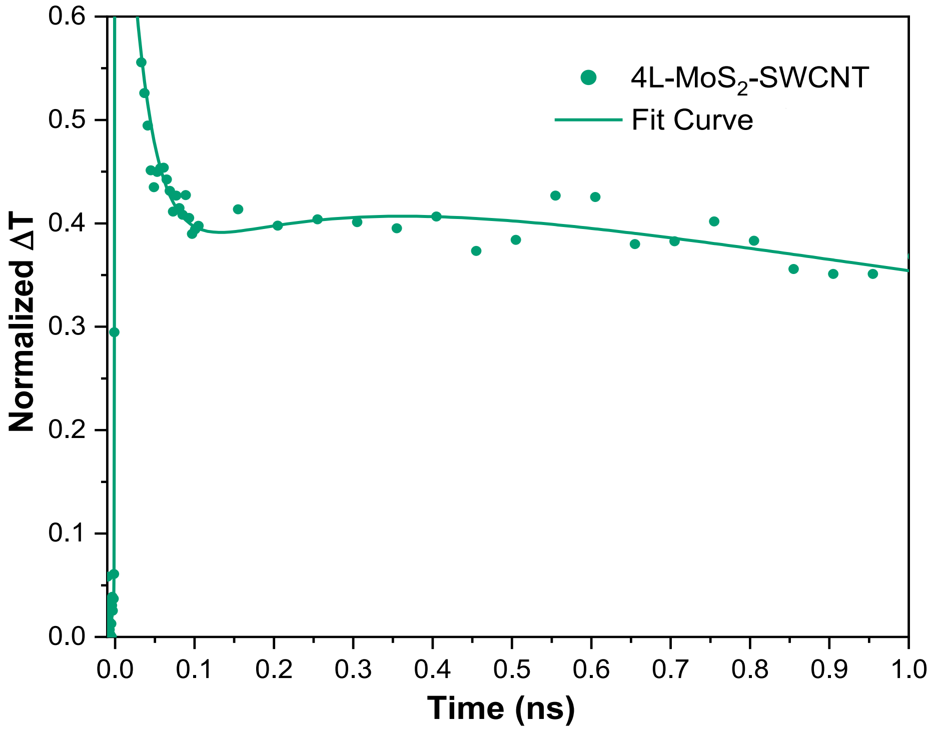

Fig. S6.

Additional rise time in nL MoS_2-SWCNT heterostructures. TA dynamics probed at the A exciton bleach of the neat MoS_2 and MoS_2 in a heterostructure for 4L- MoS_2 in the 1 ns time range. The fitting parameters are given in Table S1.

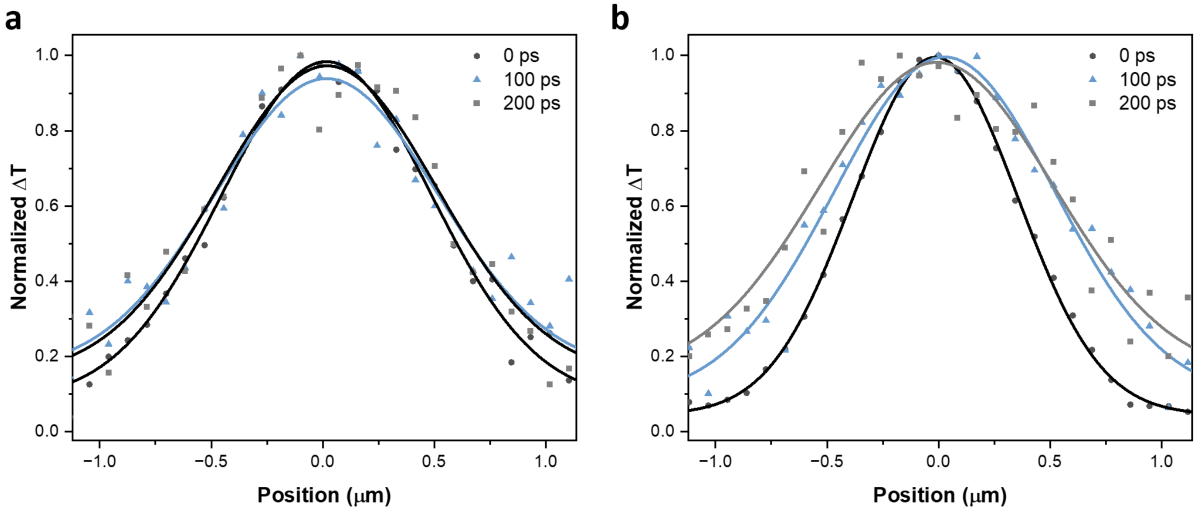

Fig. S7. Carrier diffusion in 3L- and 5L-MoS_2-SWCNT. Carrier population profiles in the (a) 3L- and (b) 5L-MoS_2-SWCNT heterostructure fitted with Gaussian functions, with the maximum ΔT signal normalized, taken at 0 ps, 100 ps, and 200 ps time delay between the pump (820 nm) and probe (670 nm). The exciton density was fixed at $5.0 \times 10^{12}\ cm^{-2}$.

.

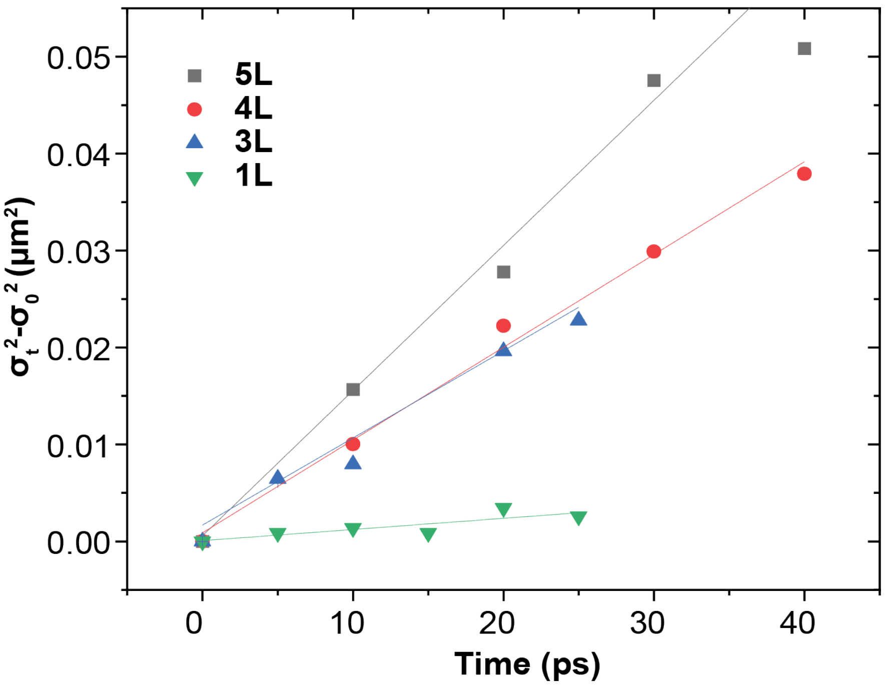

Fig. S8.

Thickness-dependent exciton diffusion in control MoS_2 Layers. $\sigma_t^2 - \sigma_0^2$ as a function of delay time for the controlled 1L, 3L, 4L and 5L-MoS_2.

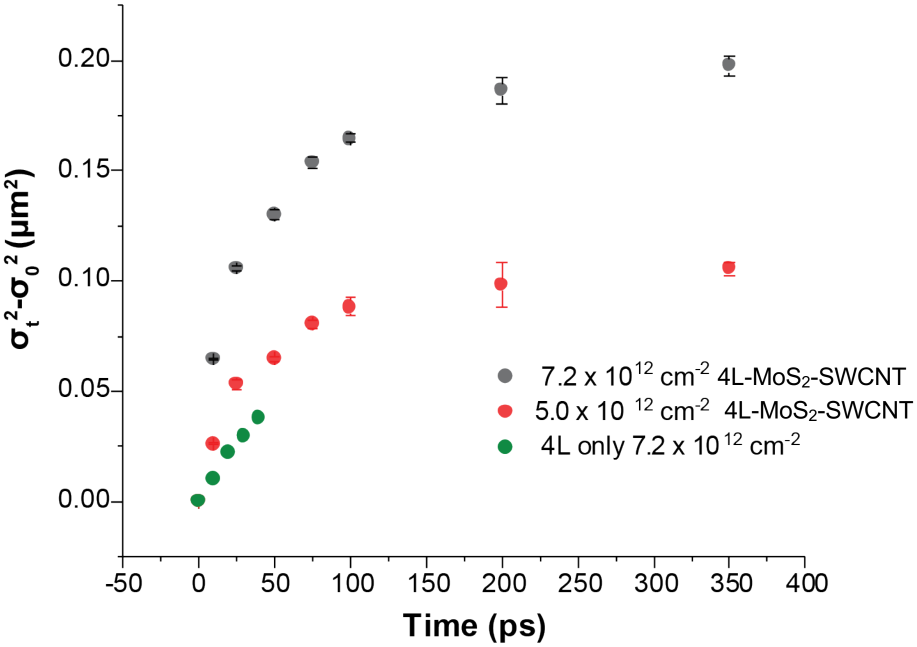

Fig. S9.
Comparing diffusion in the 4L-MoS_2-SWCNT heterostructure and the control 4L-MoS_2 sample. Exciton density-dependent $\sigma_t^2 - \sigma_0^2$ as a function of delay time in the 4L-MoS_2-SWCNT heterostructure and the controlled 4L-MoS_2.

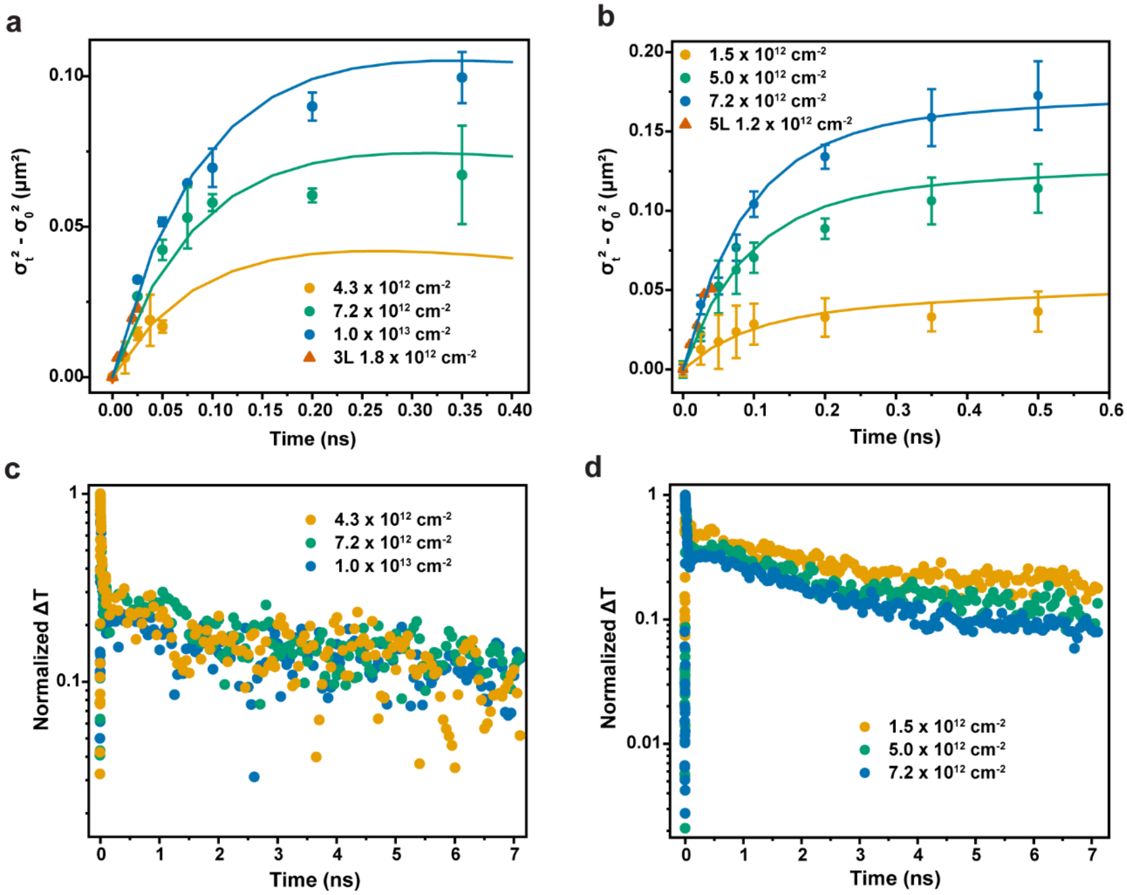

Fig. S10.

Diffusion in 3L- and 5L-MoS$_2$-SWCNT heterostructures. Exciton density-dependent $\sigma_t^2 - \sigma_0^2$ as a function of delay time in the (a) 3L-MoS$_2$-SWCNT and (b) 5L-MoS$_2$-SWCNT heterostructure. The solid lines are fits using Eq. 2. The carrier transport in the 3L- and 5L-MoS$_2$-SWCNT heterostructures is also compared with the exciton diffusion in 3L- and 5L-MoS$_2$ excited at 630 nm and probed at 670 nm. Exciton density-dependent carrier dynamics after 820 nm excitation of the (c) 3L-MoS$_2$-SWCNT and (d) 5L-MoS$_2$-SWCNT heterostructure probed at 670 nm.

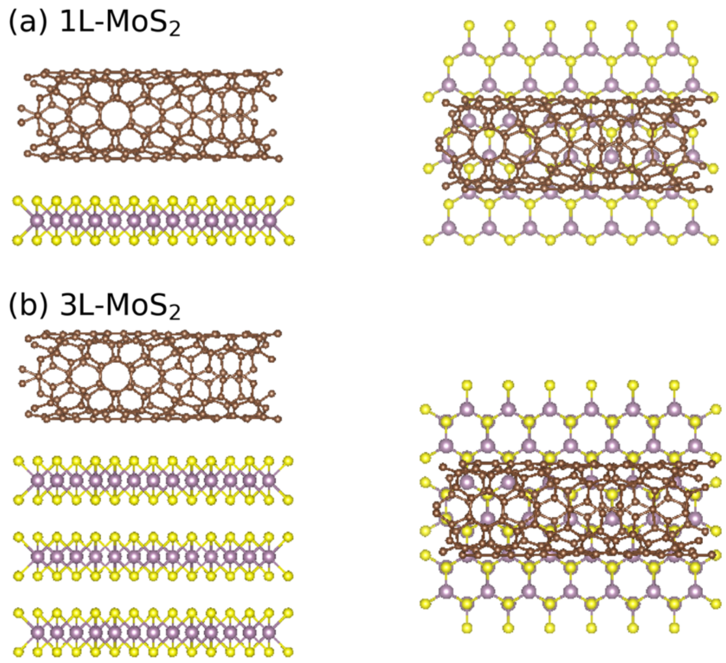

Fig. S11.
The optimized structures of **(a)** 1L- and **(b)** 3L-MoS_2 interfaced with the SWCNT.

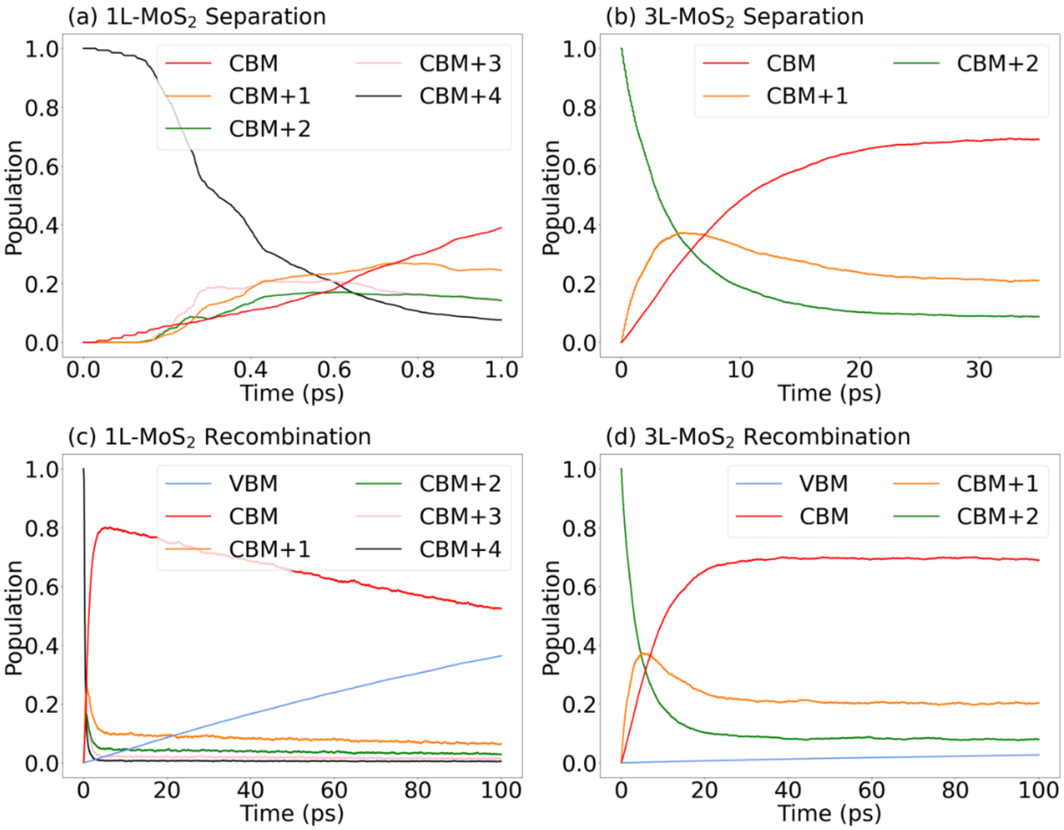

Fig. S12.
Populations of key energy levels during **(a, b)** charge separation and **(c, d)** charge recombination in the 1L- and 3L-MoS_2 systems that are interfaced with a SWCNT. In 1L-MoS_2-SWCNT, the VBM and CBM+4 are localized on the SWCNT, while the CBM, CBM+1, CBM+2 and CBM+3 are localized on MoS_2. In 3L-MoS_2-SWCNT, the VBM and CBM+2 are localized on the SWCNT, while the CBM and CBM+1 are on MoS_2. The corresponding electronic densities of states are shown in Figure 4a,b. The populations of the initial states for charge separation, i.e., CBM+4 for the 1L- and CBM+2 for the 3L-MoS_2 systems, as well as the populations of the final states for the charge recombination, i.e., the VBM, in the two systems are compared in Figure 4c,d.

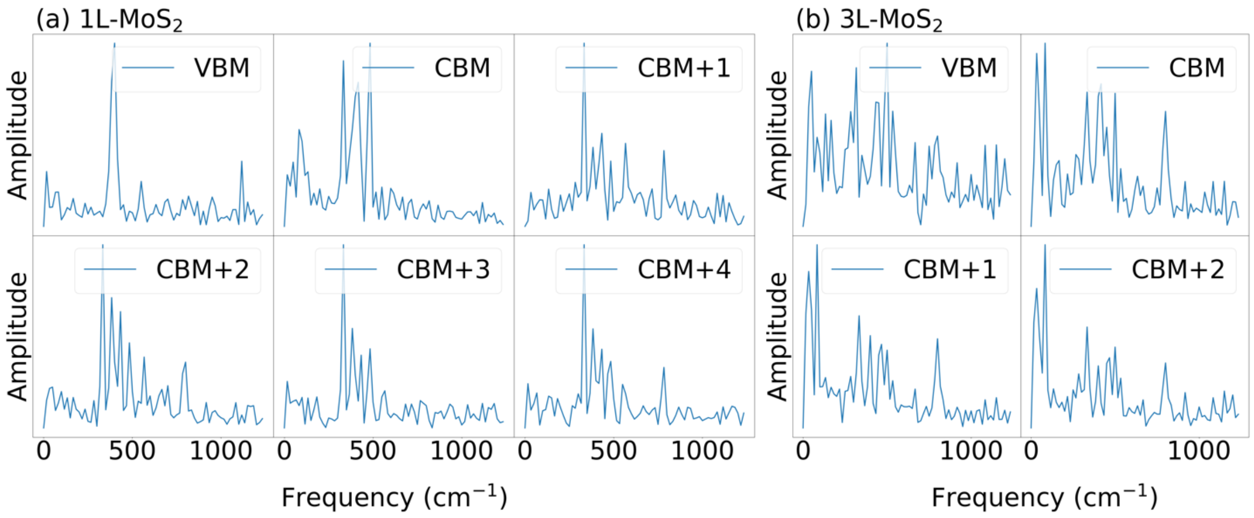

Fig. S13.
Fourier transforms (FTs) of phonon-induced fluctuations of the electronic energy levels in **(a)** 1L- and **(b)** 3L-MoS_2 interfaced with the SWCNT at 300 K. FTs of the VBM-CBM energy gap are shown in Figure 4e. The 3L system exhibits more signals in the low frequency range than the 1L system.

Table S1. In Fig. 2b-d of the main text, the dynamics were fit using the following multi-exponential function convoluted with a Gaussian response function (with the width of w)

$$y = A\exp\left(\frac{w^2-2t_1x+2t_1x_0}{2t_1^2}\right)\left(1-erf\,\frac{w^2-2t_1x+2t_1x_0}{\sqrt{2}wt_1}\right) + \mathrm{B}\ \exp\left(\frac{w^2-2t_2x+2t_2x_0}{2t_2^2}\right)\left(1-erf\,\frac{w^2-2t_2x+2t_2x_0}{\sqrt{2}wt_2}\right) + C\exp\left(\frac{w^2-2t_3x+2t_3x_0}{2t_3^2}\right)\left(1-erf\,\frac{w^2-2t_3x+2t_3x_0}{\sqrt{2}wt_3}\right)$$

The fitting parameters are summarized below:

**Samples**	**A**	**t_1 (ps)**	**B**	**t_2 (ps)**	**C**	**t_3 (ns)**
4L-MoS_2-SWCNT	0.33	29 ± 2	-0.09 (rise)	248 ± 80 (rise time)	0.2	2.1 ± 0.2
4L-MoS_2	0.42	16 ± 0.5	0.05	440 ± 160	-	-
1L-MoS_2-SWCNT	0.35	33 ± 3	0.24	540 ± 120	-	-
1L-MoS_2	1	37 ± 3	-	-	-	-

Table S2. Dipole moments of the charge separated state in the direction perpendicular to the interface, NAMD times for charge separation and recombination, root-mean square NA coupling between VBM and CBM in the interfaces composed of the SWCNT and 1L- or 3L- MoS_2.

	Dipole Moment (eÅ)	Separation Time (ps)	Recombination Time (ps)	NA Coupling (meV)
1L MoS2-SWCNT	0.843	0.310	259.5	0.220
3L MoS2-SWCNT	2.315	5.890	3514	0.0763
